# Polycarboxyl Fluorescent Hydrogel Prepared via Rapid Co-Crosslinking of Amino Carbon Dots and Composite Polysaccharide for Cr(VI) Detection and Efficient Adsorption of Neutral Red

**DOI:** 10.3390/ma19214750

**Published:** 2026-11-09

**Authors:** Yichao Wang, Chunrong Qin, Rui Luo, Sihua Zeng

**Affiliations:** Guangxi Key Laboratory of Calcium Carbonate Resources Comprehensive Utilization, College of Materials and Chemical Engineering, Hezhou University, Hezhou 542899, China; 13338329288@163.com (Y.W.); 14777562116@163.com (C.Q.); 18278236440@163.com (R.L.)

**Keywords:** aminated carbon dots, Schiff base crosslinking, fluorescent hydrogel, Cr(VI) detection, neutral red adsorption

## Abstract

Multifunctional hydrogels integrating fluorescence sensing and adsorption have application potential in water treatment, but often face the problem of overlap between crosslinking sites and functional sites. To address this, a “functional differentiation of groups” strategy is proposed in this study. Using carboxymethyl chitosan (CMCS) as the precursor, aminated carbon dots (CD-NH_2_) were synthesized by a hydrothermal method, and CMCS was used as the gel backbone. Using oxidized sodium alginate (OSA) as an aldehyde crosslinker, a fluorescent composite hydrogel (CD–gel) was prepared within 10 min via a room-temperature aqueous Schiff base reaction. In this network, –NH_2_ participates in Schiff base crosslinking, while –COOH is retained to provide adsorption sites; CD-NH_2_ are covalently anchored in the network, endowing the gel with fluorescence responsiveness. FTIR and XPS results support the formation of the Schiff base crosslinked network and the retention of carboxyl groups. SEM and N_2_ adsorption–desorption measurements show that the gel has a honeycomb-like meso–macroporous structure and is negatively charged over a broad pH range. The gel exhibits a fluorescence quenching response to Cr(VI) with a linear range of 0–80 μM (R^2^ = 0.990), a detection limit of 0.226 μM, rapid response, and anti-interference ability. Meanwhile, the gel exhibits rapid and efficient adsorption toward the cationic dye neutral red (NR), and the adsorption process follows the pseudo-second-order kinetic equation and the Langmuir isotherm equation, with a maximum theoretical adsorption capacity of 739.13 mg g^−1^. This study provides a group-engineering idea for the design of multifunctional hydrogels.

## 1. Introduction

Hydrogels are three-dimensional porous soft materials constructed from hydrophilic polymer chains through physical or chemical crosslinking. With tunable network structures, abundant chemical modification sites, and generally certain aqueous stability and separability, they are considered one of the promising candidate materials in the field of wastewater treatment [1,2,3]. However, many traditional hydrogels have relatively limited functionality, making it difficult to integrate multiple functions such as fluorescence response, catalysis, and adsorption within a single network, which restricts their multifunctional integrated applications in complex water treatment scenarios. Introducing functional nanomaterials into the gel network to construct nanocomposite hydrogels is expected to endow the gel system with new functional units and promote functional synergy, representing an important strategy for expanding the water treatment applications of hydrogels [4,5].

Carbon dots (CDs) are a class of zero-dimensional carbon nanomaterials, typically smaller than 10 nm. Owing to their quantum confinement effect, tunable fluorescence emission, excellent photostability, and low biotoxicity, CDs have been widely used as fluorescent signal units [6,7]. However, when used alone, CDs readily undergo aggregation-induced quenching, and their nanoscale dimensions make them difficult to recover from the liquid phase, limiting their practical applications. Introducing CDs into a three-dimensional hydrogel network can, on the one hand, suppress their aggregation through spatial isolation, and on the other hand, endow the composite with a macroscopic bulk form that facilitates separation and recovery. More importantly, the introduction of CDs can provide fluorescence responsiveness to traditionally non-fluorescent gel frameworks, thereby enabling pollutant recognition and enrichment on the same material platform [8,9,10]. Based on this synergistic advantage, CD–hydrogel composites have been widely studied for the detection and adsorption of heavy metal ions and organic pollutants [11,12]. These works provide important references for the functional design of CD–hydrogels, but they have also gradually revealed the inherent contradiction between crosslinking chemistry and functional sites [13].

In the construction of CD–gel composites, physical blending mainly relies on noncovalent interactions, but immobilized CDs readily leak, leading to unstable fluorescence signals [14,15]. Chemical crosslinking can anchor CDs within the network through covalent bonds, reducing leakage and migration and improving structural stability [16,17,18]. However, to achieve chemical crosslinking of CDs, the CD surface usually needs to be pre-functionalized, for example through amination, carboxylation, or the introduction of crosslinkable groups. These post-modification steps increase fabrication complexity and may affect the fluorescence properties of CDs [19]. Among these approaches, the Schiff base reaction between aldehyde and amino groups proceeds under mild conditions and can be carried out in the aqueous phase at room temperature making it a common route for immobilizing CDs [20]. However, this system generally faces a structural contradiction: reactive groups such as amino groups on the gel backbone and CD surface are consumed during crosslinking, and these groups are often also active sites for adsorption or recognition. The overlap between crosslinking sites and functional sites may limit the adsorption capacity while enabling the material to acquire fluorescence responsiveness. This structure–function contradiction restricts the degree of integration of multifunctional hydrogels [21].

**Figure 1:**
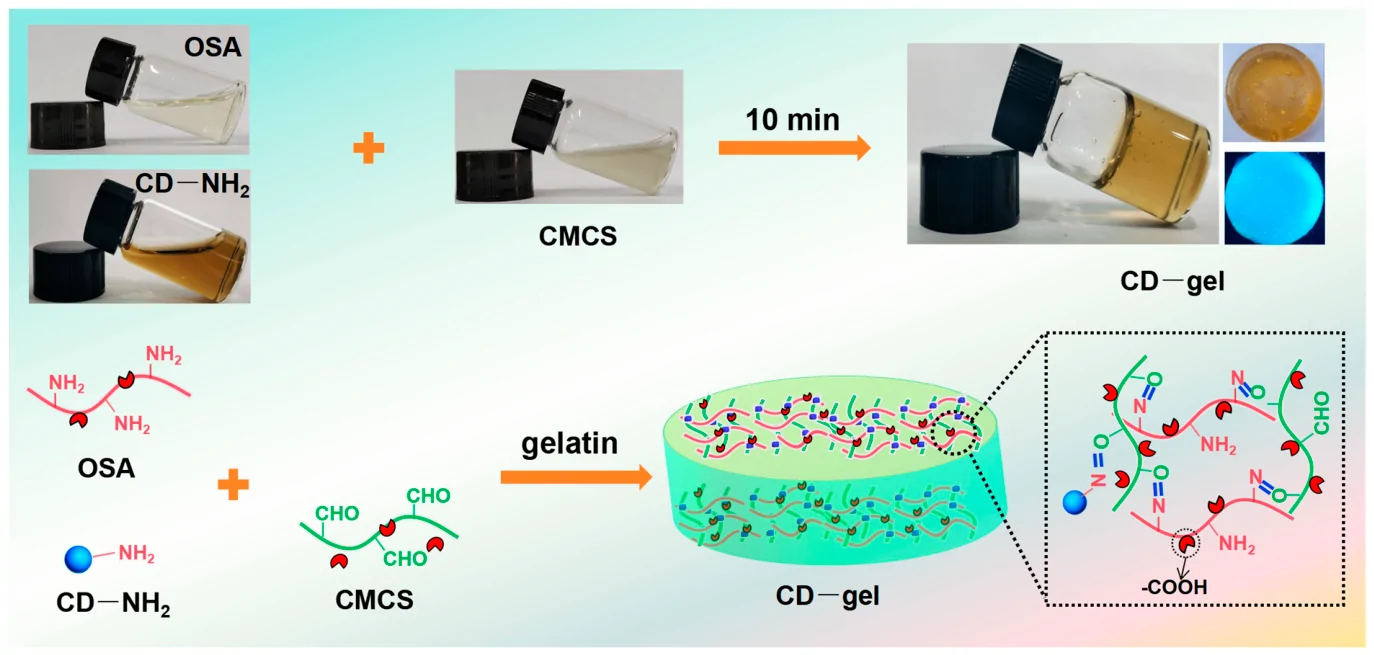
Schematic illustration of CD–gel.

To address the above contradiction, this study attempts to construct a gel network from the perspective of group division of labor and proposes a design concept of “functional differentiation of groups.” Specifically, carboxymethyl chitosan (CMCS) was used simultaneously as the carbon source and the amino source to prepare aminated carbon dots (CD-NH_2_) in situ via a hydrothermal method, without adding additional amine-containing small molecules or performing post-amination modification of the carbon dots. Subsequently, oxidized sodium alginate (OSA) was employed as an aldehyde crosslinker; its –CHO groups underwent Schiff base reactions with the –NH_2_ groups on the CMCS backbone and the CD-NH_2_ surface, enabling chemical crosslinking under room-temperature aqueous conditions and covalently incorporating CD-NH_2_ into the gel network rather than merely fixing them by physical blending. In this network, the –NH_2_ groups mainly participate in the crosslinking reaction, while the –COOH groups not involved in crosslinking are retained as much as possible. Thus, the crosslinking sites and functional sites are differentiated in design: CDs are covalently anchored as fluorescent signal units, endowing the gel with fluorescence responsiveness, while the retained –COOH groups provide functional sites for the adsorption of cationic dyes. To preliminarily examine the functional properties of the gel under this design concept, Cr(VI) fluorescence detection and NR adsorption were used as two functional equations, in which Cr(VI) was used to demonstrate the fluorescence response of the gel and NR to demonstrate its adsorption capacity. This work provides a new idea based on “functional differentiation of groups” for the rational design of multifunctional hydrogels (shown in Figure 1).

## 2. Materials and Methods

### 2.1. Materials

Sodium alginate (SA), carboxymethyl chitosan (CMCS), copper chloride, potassium chloride, calcium chloride, cadmium chloride, manganese chloride tetrahydrate, silver nitrate, anhydrous zinc sulfate, potassium dichromate, cobalt sulfate hydrate, ferric chloride hexahydrate, nickel nitrate hexahydrate, and ethylene glycol were all of analytical reagent (AR) grade and were purchased from Shanghai Aladdin Reagent Co., Ltd.; sodium periodate and NR were of AR grade and were purchased from Beijing Innochem Technology Co., Ltd., Shanghai, China; sodium hydroxide and hydrochloric acid were both of AR grade and were produced by Xilong Scientific Co., Ltd., Shantou, China; sodium chloride (AR) was provided by Shenyang Dongxing Reagent Factory, Shenyang, China; ethanol (AR) was purchased from Sinopharm Chemical Reagent Co., Ltd., Shanghai, China. Deionized water was used in the experiments.

### 2.2. Characterization

Sample morphology was examined by field-emission scanning electron microscopy (SEM, S4800, Hitachi, Tokyo, Japan) after the specimens had been lyophilized. FTIR spectra were recorded on a Thermo Nicolet Nexus 470 spectrometer (Thermo Fisher Scientific, Waltham, MA, USA) using KBr pellets, covering the range of 400–4000 cm^−1^. The specific surface area and pore structure were determined by N2 adsorption–desorption measurements on a JW-BK200C analyzer (JWGB Sci.&Tech., Beijing, China) using the static volumetric method. The crystalline phases were analyzed with a Rigaku UltimaIVTM 3KW X-ray diffractometer (Rigaku Corporation, Tokyo, Japan). The surface elemental composition and chemical states were probed by XPS on a Kratos Axis UltraDLD instrument (Kratos Analytical Ltd., Manchester, UK) with monochromatic Al Kα X-rays (1487.71 eV) operating at 75 W. Zeta potentials were determined with a Malvern nanoparticle and zeta potential analyzer (Malvern Panalytical Ltd., Malvern, UK) at ambient temperature within the pH range of 3–11. UV–Vis absorption spectra of the dye solutions were obtained before and after adsorption using a Shimadzu UV–Vis spectrophotometer (Shimadzu Corporation, Kyoto, Japan). Fluorescence spectra were recorded at 25 °C using a VARIAN spectrofluorometer (Varian Inc., Palo Alto, CA, USA).

### 2.3. Preparation of CD-NH_2_

Dissolve 0.5 g of CMCS in 25 mL of deionized water and stir magnetically at room temperature until completely dissolved. Transfer the resulting solution to a 50 mL polytetrafluoroethylene-lined high-pressure autoclave, add 5 mL of anhydrous ethanol, purge with argon for 5 min to remove the air inside the autoclave, and then seal it immediately. Place the sealed autoclave in a forced-air drying oven at 180 °C for 10 h. After naturally cooling to room temperature, take out the reaction product, centrifuge at 10,000× *g* for 15 min, and carefully collect the supernatant. Transfer the supernatant to a dialysis bag with a molecular weight cutoff of 500 Da, seal it, and dialyze against deionized water for 24 h, changing the external solution every 6 h. After dialysis, the purified amino-containing quantum dot solution (designated CD-NH_2_) is obtained and stored in a refrigerator at 4 °C for later use.

### 2.4. Synthesis of OSA

SA (5 g) was dissolved in deionized water (200 mL) with magnetic stirring until completely dissolved. Under light-protected conditions (the entire beaker was wrapped in aluminum foil), sodium periodate (4 g) was added to the solution, and the mixture was stirred at room temperature for an additional 5 h. After the reaction, ethylene glycol (5 mL) was added to terminate the oxidation reaction, and stirring was continued for 0.5 h. Subsequently, 3–5 volumes of ice-cold ethanol were added to the mixture to precipitate the oxidized product by ethanol precipitation. The precipitate was collected by centrifugation and washed repeatedly 3–5 times with an ethanol–water mixture (3:1, *v*/*v*) to thoroughly remove unreacted sodium periodate and other water-soluble impurities. The purified product was frozen at −20 °C for 24 h and then freeze-dried to obtain OSA powder.

### 2.5. Preparation of CD–Gel

OSA (1 g) was weighed and dissolved in deionized water (10 mL) with magnetic stirring until completely dissolved to prepare an OSA solution with a mass fraction of approximately 10%. Separately, CMCS (1 g) was weighed and dissolved in deionized water (20 mL). After complete dissolution, 2 mL of a 1 mg/mL CD-NH_2_ solution was added, and the mixture was stirred thoroughly to ensure uniform mixing. The above CMCS mixture was then mixed with the OSA solution, and stirring was continued until gelation occurred, thereby yielding a fluorescent polysaccharide composite gel designated as CD–gel. The resulting wet CD–gel was used directly for the Cr(VI) detection experiments, while a portion of the gel was freeze-dried and then used for the subsequent NR adsorption experiments.

### 2.6. Fluorescence Detection of Cr(VI)

CD–gel was immersed in Cr(VI) solutions of different concentrations (0–80 μM) and shaken for 1 min, after which the emission spectra were recorded under excitation at 350 nm. Under identical conditions, the fluorescence spectra of CD–gel were measured in the presence of 80 μM of different metal ions to evaluate its specificity. In addition, 80 μM Cr(VI) was added in the coexistence of the above metal ions, and the fluorescence quenching spectra were recorded to assess its anti-interference ability.

### 2.7. NR Adsorption Experiments

A total of 5 mg of dried CD–gel was accurately weighed into a beaker, and 5 mL of NR solution was added. Adsorption experiments were conducted under constant-temperature magnetic stirring. In the single-factor experiments, the NR concentration was fixed at 1000 mg/L, and with pH 7.0 and 288K as the baseline, the effects of pH (3, 5, 7, 9, 11), temperature (288, 308, 338 K), and contact time (0, 5, 10, 20, 30, 60, 90, 120 min) were investigated separately. In the isotherm experiments, other conditions were kept constant, and the initial NR concentration was varied from 100 to 1000 mg/L. Samples were collected at preset time intervals, centrifuged or filtered, and the supernatant concentration was measured by UV–Vis spectrophotometry at the maximum absorption wavelength of NR.

The adsorption capacities were calculated according to Q_t_ = (C_0_ − C_t_)V/m; and Q_e_ = (C_0_ − C_e_)V/m, where Qe is the equilibrium adsorption capacity, Qt is the adsorption capacity at time t, C_0_, C_t_ and C_e_ are the initial, time t, and equilibrium concentrations, respectively, V is the solution volume, and m is the mass of the CD–gel.

The thermodynamic, kinetic, and isotherm data were fitted using the Van’t Hoff equation, pseudo-first-order/pseudo-second-order/intraparticle diffusion equations, and Langmuir, Freundlich, and Temkin equations, respectively, to explore the adsorption mechanism. The equation equations, parameters, and calculation details are provided in the Supporting Information.

### 2.8. Regeneration and Reusability Experiments

To evaluate the reusability and practical application potential of the CD–gel, consecutive adsorption–desorption cycle experiments were conducted. After reaching adsorption equilibrium, 100 mg of the NR-loaded CD–gel was collected. The gel was then successively washed multiple times with ethanol and 1 M NaCl solution until the eluate became colorless, followed by thorough rinsing with deionized water to remove residual salts. Subsequently, the washed CD–gel was freeze-dried and directly used for the next adsorption cycle. This adsorption–desorption process was repeated for three consecutive cycles to assess the structural stability and reusability of the material.

### 2.9. Data Processing

All experimental results are expressed as averages obtained from three replicate runs. Origin 2018 was employed to fit the kinetic and isotherm equations. XPS data were processed and evaluated using Avantage software 6.9.

## 3. Results and Discussion

### 3.1. Characterization of CD-NH_2_

**Figure 2:**
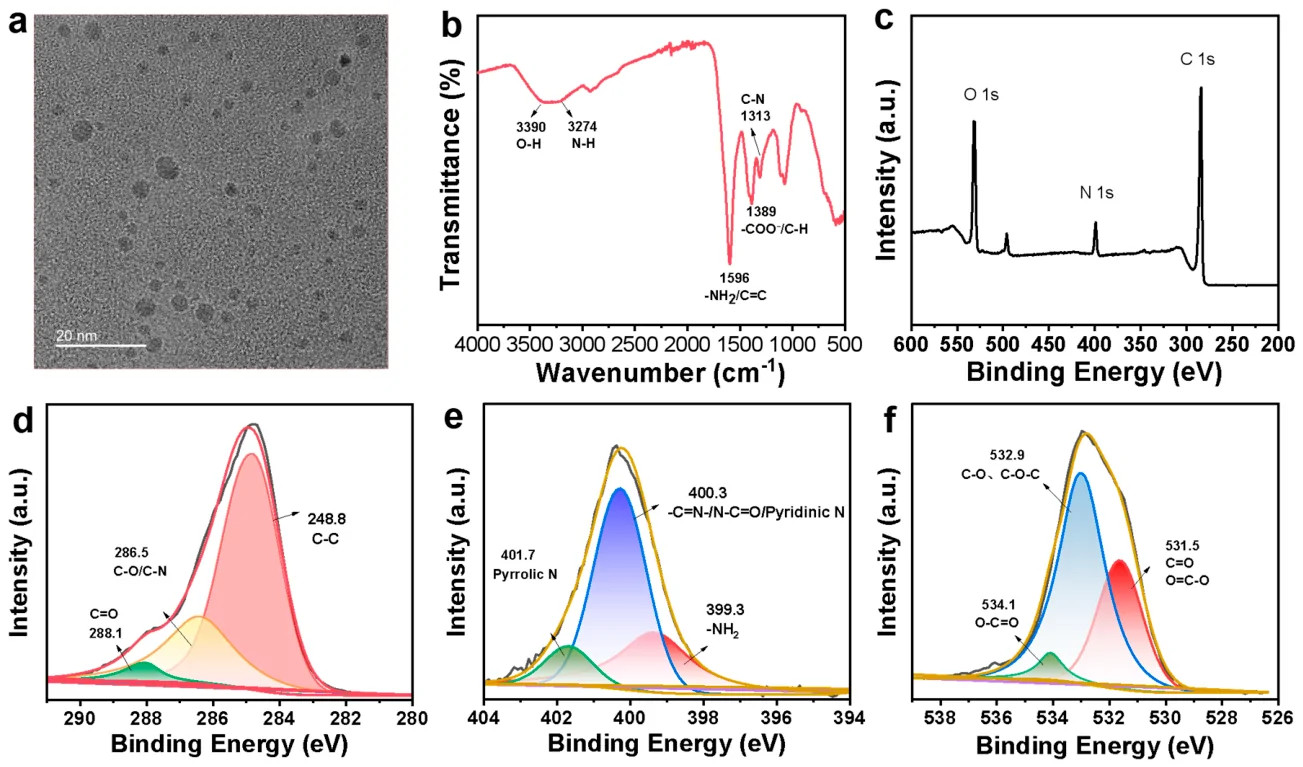
Characterization of CDs–NH_2_. (**a**) TEM image; (**b**) FTIR spectrum; (**c**) XPS survey spectrum; (**d**) high-resolution C 1s spectrum; (**e**) high-resolution N 1s spectrum; (**f**) high-resolution O 1s spectrum.

**Figure 3:**
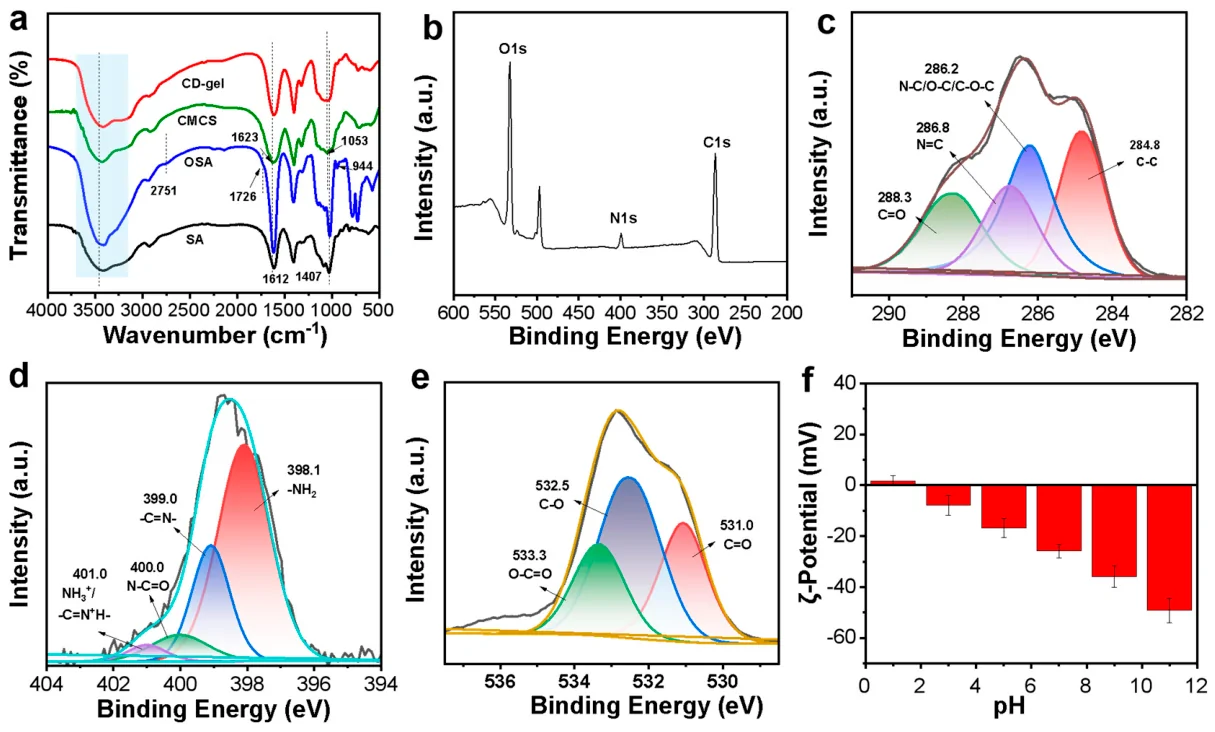
Characterization of CD–gel: (**a**) FTIR spectrum; (**b**) XPS survey spectrum; (**c**) high-resolution C 1s XPS spectrum; (**d**) high-resolution N 1s XPS spectrum; (**e**) high-resolution O 1s XPS spectrum; and (**f**) zeta potential as a function of pH.

Using CMCS as the precursor, CD-NH_2_ were prepared via a hydrothermal reaction. First, their morphology was characterized by TEM. As shown in Figure 2a, the obtained CDs had sizes ranging from 1 to 5 nm and exhibited good dispersion, and they emitted bright blue fluorescence under irradiation with a 365 nm UV lamp, indicating their excellent luminescent properties. Subsequently, FTIR (Figure 2b) was used to analyze their surface chemical groups: a broad absorption peak appeared at 3390–3274 cm^−1^, assigned to O–H/N–H stretching vibrations, indicating that the product contained hydroxyl and amino groups; the strongest absorption peak at 1596 cm^−1^ could be attributed to the bending vibration of –NH_2_ or the C=C stretching vibration of aromatic rings newly formed after hydrothermal carbonization; the absorption peak at 1389 cm^−1^ originated from the symmetric stretching vibration of carboxylate ions (–COO^−^) and the symmetric deformation vibration of methyl groups (–CH_3_). Compared with CMCS (Figure 3a), CD-NH_2_ showed decreased intensity of the O–H/N–H absorption peak and enhanced aromatic C=C stretching signal at 1596 cm^−1^, indicating that hydrothermal carbonization increased the degree of graphitization while still retaining the functional groups characteristic of the CMCS polymer [22,23]. The surface chemical composition and valence states were further analyzed by XPS. The survey scan (Figure 2c) indicated that the sample was mainly composed of three elements: C (72.3%), O (20.7%), and N (7.0%). The high-resolution C 1s spectrum (Figure 2d) could be fitted into three peaks, among which the peaks at 284.8, 286.5, and 288.1 eV were assigned to C–C, C=N/C–O, and O–C=O/N–C=O, respectively. This indicates that carboxyl, carbonyl, and amide structures were present on the surface of the CDs, and that N was incorporated into the carbon skeleton during hydrothermal carbonization [24]. The high-resolution N 1s spectrum (Figure 2e) could be deconvoluted into multiple peaks: the peak at 398.3 eV was assigned to free amino groups (–NH_2_) not involved in crosslinking; the peak at 400.3 eV was assigned to amide nitrogen (N–C=O or –C=N–) and pyridinic N [25]; and the peak around 401.7 eV corresponded to pyrrolic N. The O 1s spectrum (Figure 2f) could also be fitted into three components, assigned to C=O (531.5 eV), C–O/C–O–C (532.9 eV), and O–C=O/adsorbed oxygen (534.1 eV), respectively [26]. The above results indicate that hydrothermal carbonization introduced aromatic carbon structures, and the product surface contained functional groups such as carboxyl, hydroxyl, and amino/amide groups, which can provide suitable sites for subsequent crosslinking reactions.

### 3.2. Chemical Structure of CD–Gel

The construction of the fluorescent CD–gel is based on a two-step cascade reaction involving oxidation and condensation. First, through selective oxidation by sodium periodate, aldehyde groups are generated in situ at the C2/C3 positions of the uronic acid units in the SA molecular chain, yielding a functionalized self-crosslinker, OSA. Subsequently, efficient Schiff base condensation between the aldehyde groups of OSA and the primary amino groups of CMCS and the amino groups on the CD-NH_2_ surface completes dynamic imine bond crosslinking within 10 min at room temperature, realizing covalent integration of the macromolecular network and fluorescent carbon dots and constructing a CD–gel with both a three-dimensional network skeleton and fluorescence responsiveness. FTIR was used to track the synthesis process (Figure 3a). Compared with raw SA, the oxidized product SA-CHO exhibits new characteristic absorption peaks at 2751 cm^−1^ (aldehyde C–H stretching vibration), 1727 cm^−1^ (aldehyde carbonyl C=O stretching vibration), and 944 cm^−1^ (aldehyde C–H out-of-plane bending vibration), confirming that adjacent hydroxyl groups at the C_2_/C_3_ positions of the glucuronic acid units in SA were successfully oxidized to aldehyde groups. The peaks of raw CMCS at 1623 cm^−1^ and 1053 cm^−1^ are assigned to the N–H symmetric stretching vibration of primary amino groups and C–N in-plane bending vibration, respectively. After crosslinking, in the FTIR spectrum of the CD–gel, the aldehyde peaks (2751, 1727 cm^−1^) and the primary amino peak (1623 cm^−1^) are markedly weakened or even disappear, indicating that the aldehyde and amino groups effectively participated in the Schiff base reaction and were consumed, forming a dynamic imine crosslinked network [27,28]. Meanwhile, all samples retain asymmetric and symmetric carboxylate stretching peaks near 1612 cm^−1^ and 1405 cm^−1^, indicating that the gel network contains abundant carboxyl functional groups.

XPS analysis further revealed the chemical composition and bonding states of the gel surface. The survey scan (Figure 3b) showed that the sample was mainly composed of C (59.4%), N (4.41%), and O (36.1%). The high-resolution C 1s spectrum (Figure 3b) could be fitted into five peaks, located at 284.8, 286.2, 286.8, and 288.3 eV, assigned to C–C, C–N/C–O/C–O–C, C=N, and C=O, respectively. Among them, the appearance of C=N at 286.8 eV and the signal of O–C=O/N–C=O at 283.3 eV indicated that the –COOH groups of CMCS coexisted with amide bonds formed by crosslinking, and that the Schiff base reaction had occurred. The high-resolution N 1s spectrum (Figure 3c) could be deconvoluted into multiple peaks: the peak at 398.1 eV was assigned to free amino groups (–NH_2_) not involved in crosslinking, the peak at 399.0 eV was assigned to imine bonds (–C=N–), the peak at 400.0 eV was assigned to amide nitrogen (N–C=O), and the region around 401.0 eV corresponded to protonated amines (–NH_3_^+^ and –C=N^+^H–). The appearance of the characteristic imine bond peak at 399.0 eV directly confirmed that the aldehyde groups in SA-CHO underwent Schiff base condensation with the amino groups on CMCS and CD-NH_2_. The O 1s spectrum (Figure 3d) could likewise be fitted into three components, assigned to C=O (531.0 eV), C–O (532.5 eV), and O–C=O (533.6 eV), respectively, serving as supporting evidence. Combined with the appearance of the characteristic imine bond (–C=N–) peak in the N 1s spectrum and the changes in the relevant signals in the C 1s spectrum, this further corroborated the FTIR conclusions, indicating that the dynamic Schiff base crosslinked network had been successfully constructed and that CD-NH_2_ was covalently anchored in the gel framework.

Zeta potential measurements (Figure 3f) show that the CD–gel surface is negatively charged over the pH 3–11 range, with the potential gradually decreasing from 1.72 mV at pH 1 to −49.0 mV at pH 11. Its negative charge over a broad pH range originates from the contribution of numerous deprotonated carboxyl groups within the network, endowing the material with strong electrostatic affinity for cations and suggesting good application potential in the field of cationic adsorption.

### 3.3. Surface Structure of CD–Gel

**Figure 4:**
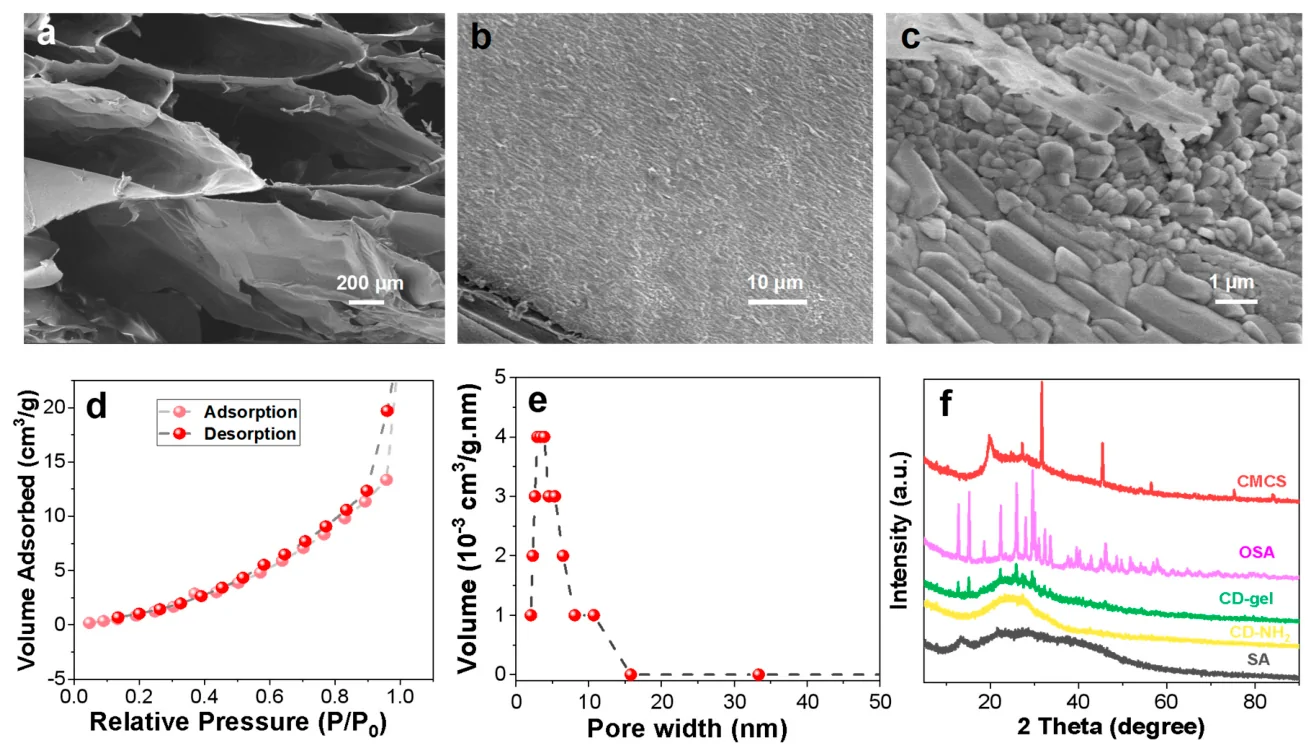
(**a**–**c**) SEM images of CD–gel at different magnifications. (**d**) N_2_ adsorption–desorption isotherm. (**e**) Pore size distribution. (**f**) XRD pattern.

To observe the micromorphology, the CD–gel was freeze-dried and then characterized by SEM. Low-magnification (50×) micrographs revealed an interconnected macroporous network in all samples (Figure 4a), indicating that effective crosslinking among polymer chains formed a three-dimensional skeleton. At higher magnifications (1000× and 10,000×, Figure 4b,c), the gel surface exhibited a rough and regular morphology with abundant pores. N_2_ adsorption–desorption measurements (Figure 4d,e) revealed that the gel exhibits a multipoint BET surface area of 61.2 m^2^·g^−1^ and an adsorption total pore volume of 0.035 cm^3^·g^−1^. Notably, the pore architecture is predominantly mesoporous, as evidenced by the BJH adsorption average pore diameter of 6.3 nm and a most probable pore size of 5.2 nm, while micropores and macropores are virtually absent. Such a mesoporous framework offers structural benefits by exposing abundant accessible active sites and facilitating the efficient diffusion of contaminants during the adsorption process.

The crystalline characteristics of SA, OSA, CMCS, CD-NH_2_, and CD–gel were investigated by XRD. For the raw materials, SA exhibited semicrystalline characteristics; OSA showed increased crystallinity after oxidative rearrangement; and CMCS appeared amorphous due to carboxymethyl substitution. In the final XRD pattern of the CD–gel, the characteristic diffraction peaks of CMCS and OSA almost disappeared, leaving only a broad diffuse halo centered at approximately 21.6°, accompanied by a marked reduction in overall diffraction intensity. This indicated that the Schiff-base crosslinking and intermolecular hydrogen bonding formed during gelation disrupted the original ordered structures of the raw materials, transforming the gel into an amorphous network. The CD–gel featured a hierarchical pore structure, a high specific surface area, and an amorphous skeleton, all of which are favorable for the mass transfer and contact of small molecules and ions, thereby providing a structural basis for its subsequent adsorption and fluorescence detection performance.

### 3.4. Detection of Cr(VI)

**Figure 5:**
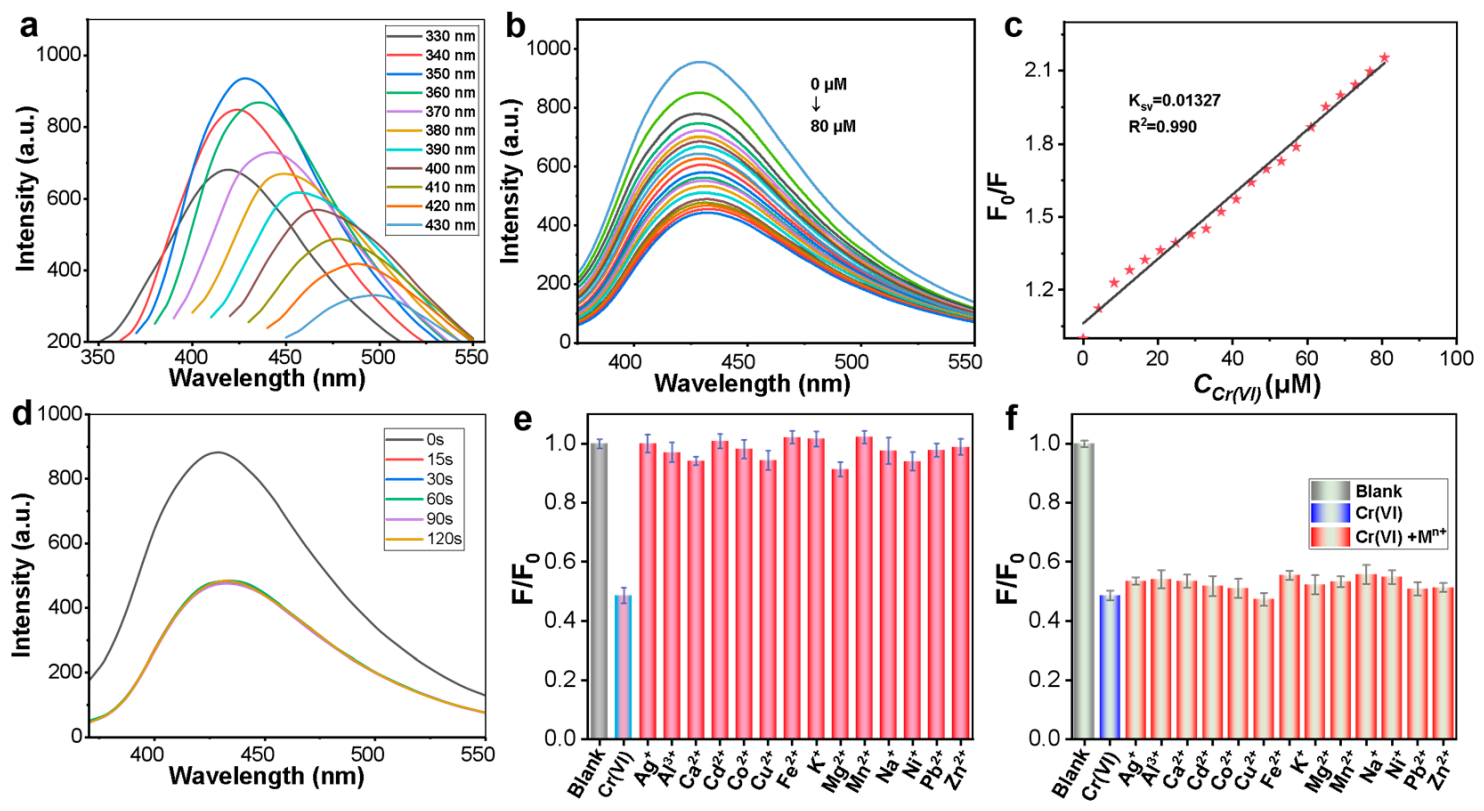
(**a**) Fluorescence emission spectra of CD–gel excited at different wavelengths. (**b**) Emission spectra of CD–gel after interaction with various concentrations of Cr(VI) (λ_ex_ = 350 nm). (**c**) Linear relationship between the fluorescence intensity ratio F/F_0_ and Cr(VI) concentration in the range of 0–80 μM, where F_0_ and F represent the fluorescence intensities of CD–gel in the absence and presence of Cr(VI), respectively. (**d**) Fluorescence emission spectra of CD–gel mixed with 80 μM Cr(VI) at different reaction times. (**e**) Relative fluorescence intensity (F/F0) of CD–gel in the presence of 80 μM different metal ions. (**f**) Comparison of the effect on the fluorescence intensity (F/F0) of CD–gel for 80 μM Cr(VI) alone and in the presence of coexisting ions.

Cr(VI) is highly toxic and poses a serious threat to aquatic environmental safety and human health; therefore, developing efficient and sensitive methods for Cr(VI) detection is of great significance [29,30,31]. In this study, the fluorescence properties of CD–gel were first characterized, and the results revealed that the material exhibits a distinct excitation-wavelength dependence [32]. As the excitation wavelength was gradually increased from 320 nm to 410 nm, the fluorescence emission peak redshifted from 380 nm to 440 nm (Figure 5a). Through optimization, the optimal excitation wavelength was determined to be 350 nm, at which the emission maximum was located at 435 nm; all subsequent detection experiments were performed under this condition.

Under the above optimized conditions, the response behavior of the CD–gel fluorescence system toward Cr(VI) was further investigated. Fluorescence spectra showed that the addition of Cr(VI) could effectively quench the characteristic fluorescence signal of CD–gel (Figure 5b). In the concentration range of 0–80 μM, the fluorescence intensity at 435 nm decreased continuously with increasing Cr(VI) concentration. Quantitative analysis revealed a good linear relationship between the fluorescence quenching ratio (F_0_/F) and Cr(VI) concentration, with a correlation coefficient R^2^ = 0.990. Based on the detection limit formula LOD = 3Sd/S, the limit of detection for Cr(VI) by this sensing system was calculated to be 0.226 μM (0.0114 mg L^−1^) (Figure 5c). Compared with conventional detection techniques and previously reported fluorescent probes, the CD–gel sensor constructed in this work exhibits distinct advantages in overall analytical performance: its detection limit is far below the safe limit for Cr(VI) in drinking water set by the World Health Organization (0.05 mg L^−1^), thus meeting the requirements for trace Cr(VI) detection in water [33]; it offers a wide linear range of 0–80 μM, and the fluorescence signal can reach stability within 15 s (Figure 5d), significantly improving detection efficiency over traditional methods [34]; moreover, the detection process does not require toxic reagents, combining good environmental friendliness and operational safety, and demonstrating considerable potential for practical applications.

Selectivity is a critical parameter for evaluating the performance of fluorescent sensing probes. In this study, several common metal ions in water, including Co^2+^, Pb^2+^, Cu^2+^, Ca^2+^, and Fe^3+^, were selected as interfering species to investigate the recognition specificity of CDs toward Cr(VI). As shown by the relative fluorescence intensity (F/F_0_) results in Figure 5e, under the same test concentration, only Cr(VI) induced a significant fluorescence quenching effect, with the relative intensity decreasing to 0.486, whereas the other metal ions had almost no effect on the probe fluorescence, indicating that the probe possesses excellent specificity for Cr(VI) recognition. Further validation was carried out through coexisting ion interference experiments, in which the fluorescence spectra of a single Cr(VI) system and a system containing Cr(VI) together with multiple interfering ions were compared (Figure 5f). The results showed that the coexisting ions caused only very weak additional fluorescence quenching, and the interference with the quantitative detection of Cr(VI) was negligible, fully demonstrating that the CD–gel probe can maintain stable and reliable detection performance even in complex water matrices.

### 3.5. Adsorption Performance of NR

**Figure 6:**
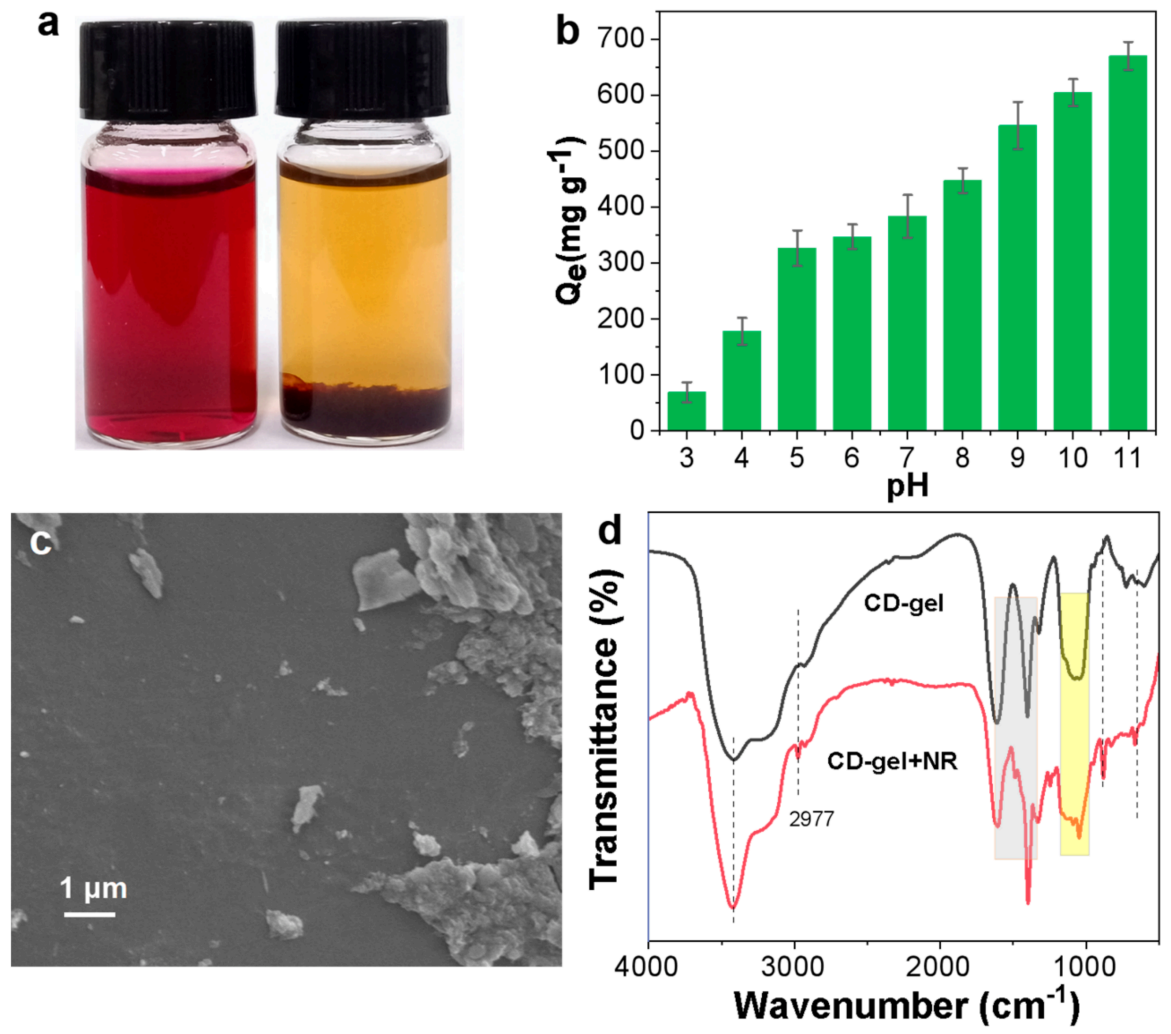
(**a**) Photos of NR solution (C_0_ = 1000 mg L^–1^) before and after 10 min adsorption with 5 mg CD–gel. (**b**) Adsorption capacity of 5 mg CD–gel for NR (C_0_ = 1000 mg L^–1^) at different pH. (**c**) SEM morphology and (**d**) FTIR spectra of the gel after adsorption.

Cationic dye NR, as a typical organic pollutant in printing and dyeing wastewater, possesses potential toxicity and bioaccumulation, posing a continuous threat to aquatic ecosystems. Developing efficient adsorbent materials is key to the treatment of this pollutant [35]. To this end, CD–gel was used as an adsorbent to systematically investigate its adsorption behavior toward NR in aqueous solution. First, the basic adsorption capacity of CD–gel was verified through preliminary experiments (Figure 6a). In total, 5mg of freeze-dried CD–gel was placed in 5 mL of NR solution (initial concentration 100 mg·L^−1^). After only 10 min of shaking, the solution showed obvious decolorization, and the gel matrix exhibited a visible red color due to NR loading, preliminarily confirming that the composite has rapid capture efficiency for NR.

Solution pH is a core parameter determining adsorption performance because it simultaneously regulates the surface charge state of the adsorbent and the existing form of dye molecules [36]. The effect of initial solution pH (3–11) on the adsorption capacity of CD–gel was systematically examined (Figure 6b). The results showed that the adsorption capacity continuously increased with increasing pH, which can be attributed to the synergistic regulation of adsorbent surface charge and dye speciation by pH. Under strongly acidic conditions (pH < 5), high concentrations of H^+^ strongly compete for the limited carboxyl binding sites, resulting in significant electrostatic repulsion between CD–gel and cationic NR molecules, thereby severely inhibiting dye anchoring. As pH increases, carboxyl groups (–COOH) in the gel skeleton gradually deprotonate to –COO^−^, the surface zeta potential becomes more negative, and the electrostatic attraction with positively charged NR molecules increases accordingly, thus greatly enhancing the adsorption capacity. Although alkaline media (pH > 9) exhibited higher adsorption capacity, considering that actual water bodies are mostly near-neutral and that strongly alkaline conditions can introduce additional operational interference, pH 7 was uniformly selected as the working medium in subsequent adsorption experiments to balance field applicability and condition universality.

To further reveal the structural response mechanism of the adsorption process, NR-loaded CD–gel was freeze-dried, and SEM and FTIR were used to characterize the micromorphology and chemical group evolution, respectively. SEM observations showed (Figure 6c) that compared with the abundant grooves and porous structure on the original gel surface, the surface roughness of the NR-adsorbed sample was significantly reduced, and the pore edges became rounded and smooth. This is attributed to NR molecules covering the original micro-/nanostructure of the gel through physical filling or surface deposition, confirming effective loading of the adsorbate. FTIR spectra (Figure 6d) provided direct evidence of adsorption at the molecular level: after adsorption, a characteristic absorption peak assigned to the C=C/C=N stretching vibrations of the NR benzene ring skeleton appeared in the 1400–1600 cm^−1^ region; meanwhile, the relative intensity of the asymmetric stretching vibration peak of carboxyl groups (–COO^−^) at ~1650 cm^−1^ decreased, indicating that carboxyl sites in the gel skeleton specifically bound to NR^+^ through electrostatic coordination or ion exchange. Notably, the C–O–C characteristic peak derived from glycosidic bonds (~1050 cm^−1^) also showed a certain degree of weakening after adsorption, suggesting that ether oxygen atoms in the gel network may act as auxiliary hydrogen-bond acceptors and participate in the synergistic capture of NR molecules. Combining the morphological and FTIR spectral evidence, the adsorption of NR by CD–gel is not merely simple surface physical enrichment, but a multi-site synergistic adsorption process dominated by electrostatic interactions and assisted by hydrogen bonding and n–π interactions. After adsorption, no significant new peaks or obvious chemical shifts appeared in the FTIR spectra, indicating that the process did not form strong covalent bonds or cause obvious chemical modification of the hydrogel structure; therefore, the binding of NR to CD–gel mainly depends on interactions involving surface active sites, such as electrostatic attraction, ion exchange/coordination, and hydrogen bonding, rather than simple diffusion or surface physical deposition. The above multiple interactions jointly contribute to the high efficiency and stability of the adsorption hydrogel.

### 3.6. Adsorption Thermodynamics, Kinetics, and Isotherms of NR

To systematically investigate the adsorption behavior of CD–gel toward NR, the adsorption isotherms, kinetics, and thermodynamics were examined in sequence.

#### 3.6.1. Adsorption Isotherms

**Figure 7:**
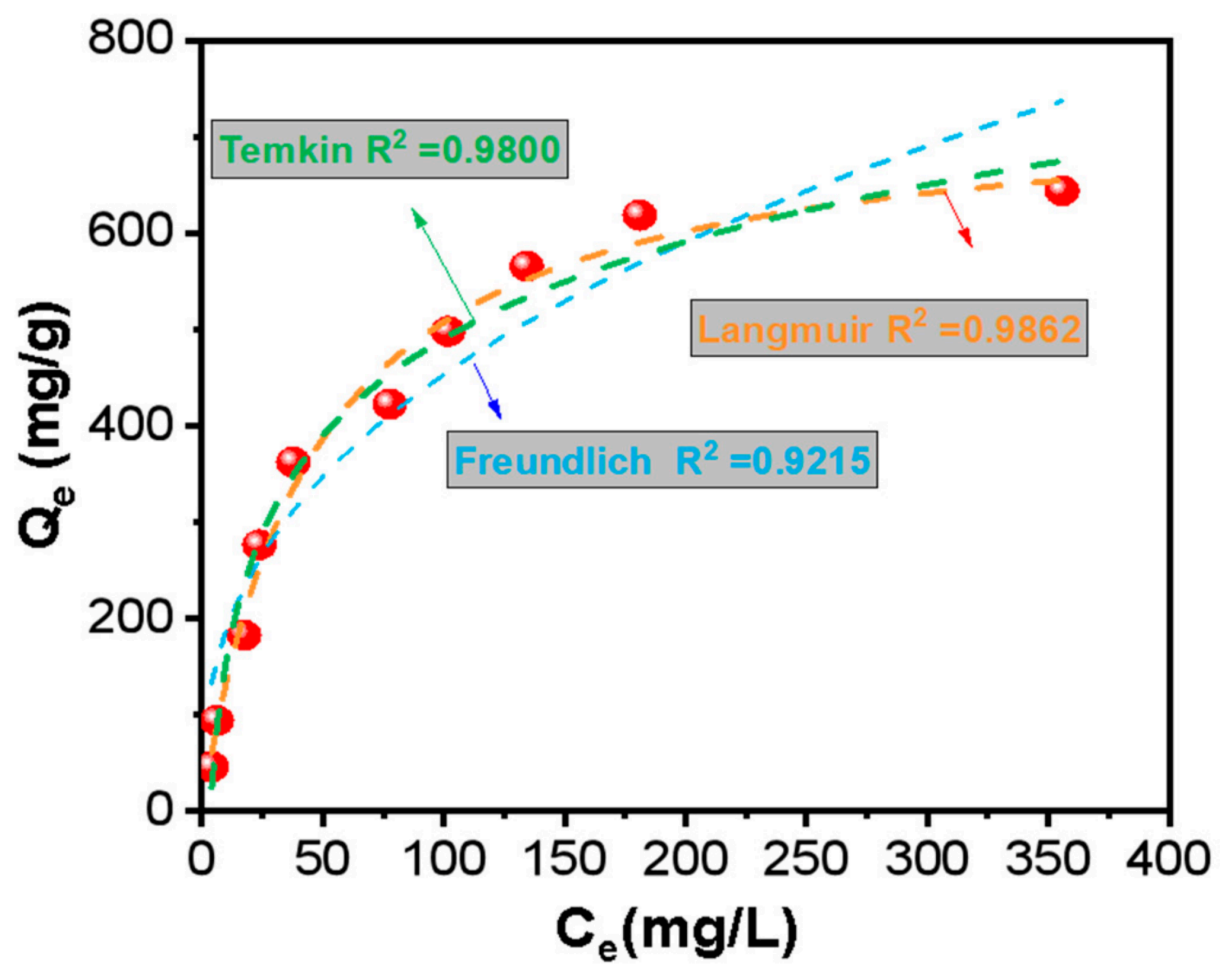
Adsorption isotherms of NR on CD–gel (288 K).

Building on the concentration gradient analysis, the adsorption behavior of NR onto CD–gel was further investigated using adsorption isotherms (Figure 7). Within the concentration range examined, the equilibrium adsorption capacity continuously increased with increasing initial concentration, indicating that the concentration gradient is an important driving force for adsorption. To clarify the adsorption mechanism, three classical equations—Langmuir, Freundlich, and Temkin—were used to fit the experimental data via non-linear regression (Figure 7 and Table 1). Among them, the Langmuir equation showed the best correlation (R^2^ = 0.9877) compared to the Temkin equation (R^2^ = 0.9800) and the Freundlich equation (R^2^ = 0.9216), suggesting that the adsorption data are consistent with monolayer adsorption on relatively homogeneous surface sites. The maximum theoretical adsorption capacity Qm of the Langmuir equation was 739.13 mg g^–1^. It is worth noting that this Qm value is higher than the experimental maximum capacity (644.27 mg g^–1^), because the highest initial concentration used in this study was not high enough to fully saturate all binding sites; Qm represents the extrapolated theoretical limit. A comparison of the maximum adsorption capacities of various reported adsorbents for NR is summarized in Table S1 [37,38,39,40,41,42,43,44,45], indicating the excellent adsorption potential of CD–gel. The Langmuir constant K_L_ = 0.0220 L mg^–1^ suggests a moderate affinity. The separation factor RL was calculated to be 0.043 at C_0_ = 1000 mg L^–1^ (0 < R_L_ < 1), further confirming favorable adsorption [46,47]. In addition, the Freundlich exponent (n = 2.61) also indicated favorable adsorption. In summary, the adsorption data are consistent with the Langmuir isotherm equation, demonstrating promising practical application potential.

#### 3.6.2. Adsorption Kinetics

**Figure 8:**
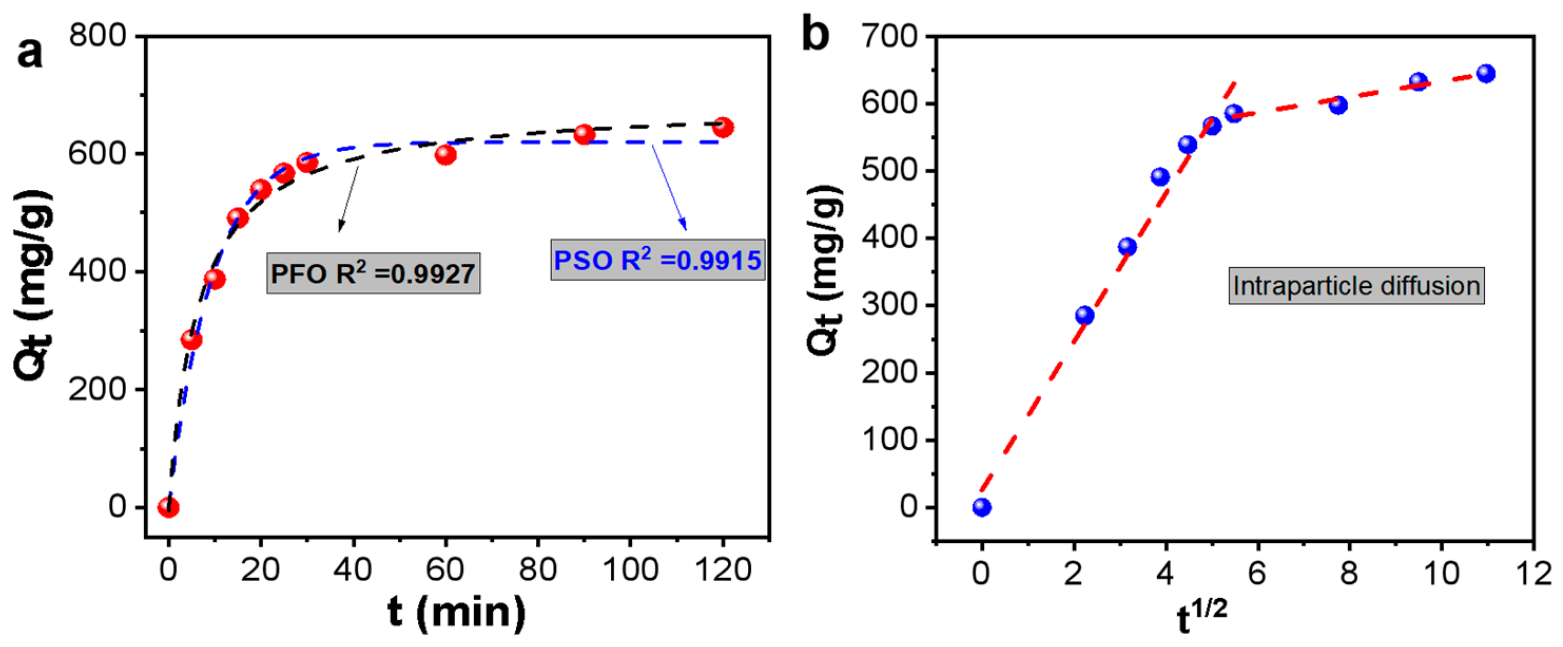
(**a**) Effect of contact time on NR adsorption (C_0_ = 1000 mg/L) and kinetic equation fitting results. (**b**) Linear relationship between t/Qt and t for pseudo-second-order kinetics.

In addition to isotherms, the adsorption rate is an important parameter for evaluating practical application potential. At pH 7 and 288 K, an initial NR concentration of 1000 mg/L was selected to investigate the effect of contact time (Figure 8). CD–gel exhibited rapid uptake in the initial stage, achieving over 60% of the equilibrium adsorption amount within the first 10 min, and reached apparent equilibrium at approximately 120 min. To elucidate the kinetic behavior, the experimental data were analyzed using pseudo-first-order (PFO), pseudo-second-order (PSO), and intraparticle diffusion equations via non-linear regression (Figure 8a and Table 2). As the system reached apparent equilibrium, the limited number of data points resulted in highly comparable R^2^ values for PFO and PSO (0.9927 and 0.9915, respectively). Therefore, relying solely on R^2^ is insufficient. From a mechanistic perspective, the adsorption of cationic NR onto anionic CD–gel is driven by strong electrostatic attractions and hydrogen bonding (chemisorption). The PSO equation provided a more realistic theoretical capacity (687.3 mg g^−1^) compared to the PFO model (619.97 mg g^−1^), which aligns with the chemisorption mechanism. Furthermore, the intraparticle diffusion plot (Figure 8b) was divided into two distinct linear stages. Stage I (0~30 min) had a steep slope (k_id1_ = 110.31 mg g^−1^ min^0.5^) and a non-zero intercept (C_1_ = 26.08 mg g^−1^), representing rapid boundary layer diffusion. Stage II (30~120 min) showed a smaller slope (k_id2_ = 11.60 mg g min^−1/2^) and a larger intercept (C_2_ = 517.5 mg g^−1^), indicating slow intraparticle diffusion. The non-zero intercepts infer that intraparticle diffusion is not the sole rate-limiting step [48,49]. Overall, chemical interactions dominate the adsorption rate.

#### 3.6.3. Adsorption Thermodynamics

**Figure 9:**
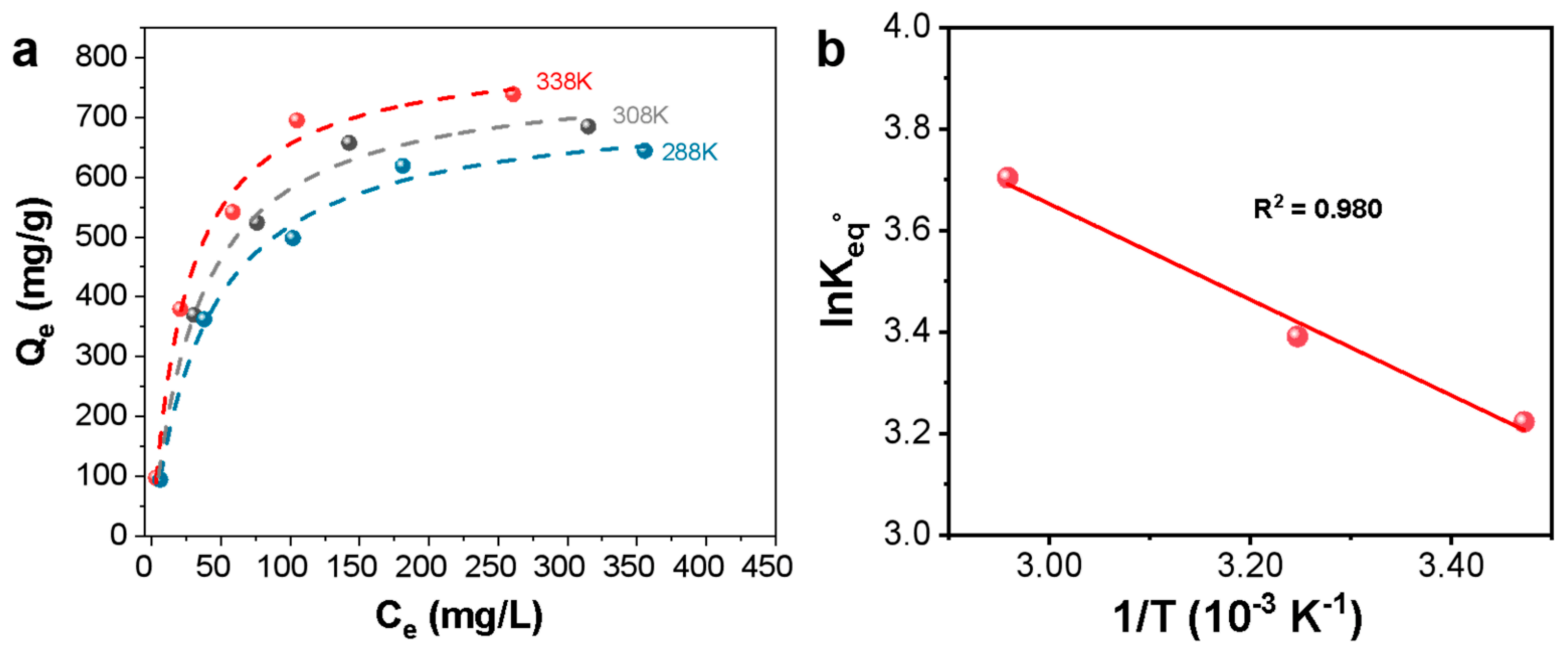
(**a**) Adsorption isotherms of NR on CD–gel at different temperatures (288 K, 308 K, and 338 K) with non-linear Langmuir fitting curves. (**b**) Van’t Hoff plot of ln Keq° versus 1/T for the adsorption of NR.

To explore the thermodynamic nature, the adsorption isotherms at 288, 308, and 338 K were compared (Figure 9a). Under the same initial concentration conditions, the equilibrium adsorption capacity gradually increased with temperature. This may be due to heating relaxing the hydrogel network, exposing more -COOH sites [50], and enhancing the thermal motion of NR, making it easier to bind. To ensure the accuracy and representativeness of the thermodynamic equilibrium constant, while the entire dataset (10 points) was used for the macroscopic isotherm model fitting (Table 3), a subset of 5 representative data points covering the low, medium, and high concentration ranges was specifically selected to calculate the Langmuir equilibrium constants (K_L_) at each temperature. The dimensionless equilibrium constant (Keq°) was derived from K_L_, as suggested by Tran et al. [51]. The Van’t Hoff plot of ln Keq° versus 1/T showed good linearity (R^2^ = 0.980; Figure 9b). The thermodynamic parameters were calculated (Table 1): ΔH° = 7.84 kJ mol^−1^ > 0, ΔS° = 53.89 J mol^−1^ K^−1^ > 0, while ΔG° at 288, 308, and 338 K was −7.68, −8.76, and −10.37 kJ mol^−1^, respectively. The positive ΔH° confirms the endothermic nature. The positive ΔS° reflects increased disorder at the solid–liquid interface. The negative ΔG° values confirm the spontaneous nature, and the increasingly negative ΔG° with temperature indicates that heating is thermodynamically favorable for adsorption [52].

### 3.7. Regeneration Performance

**Figure 10:**
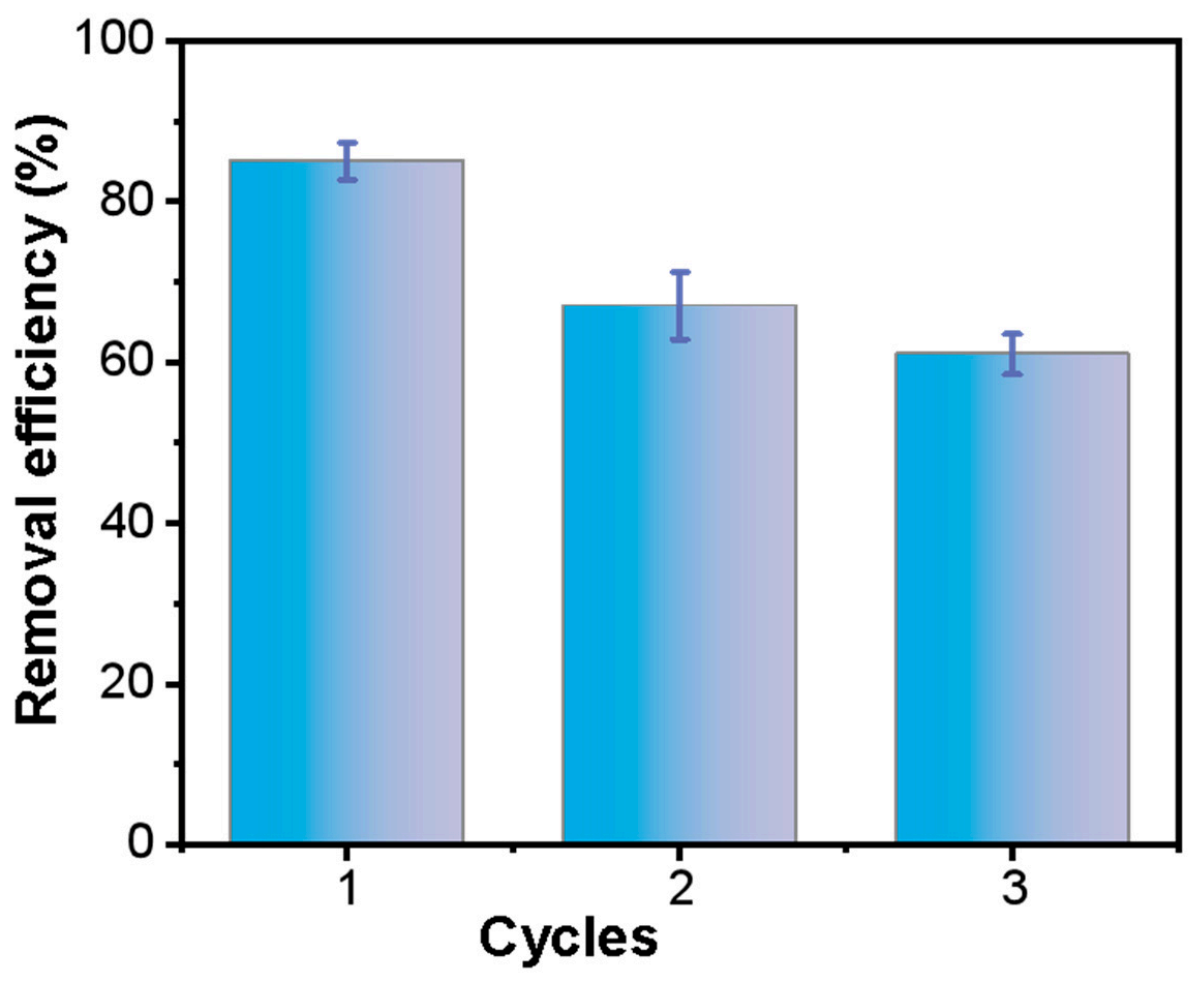
Regeneration performance of CD–gel during three consecutive cycles.

The reusability of the CD–gel was evaluated through three consecutive adsorption–desorption cycles. As shown in Figure 10, the adsorption efficiency remained at 85% after the first cycle and decreased to 67% and 61% in the second and third cycles, respectively. This decline in adsorption capacity can be attributed to the incomplete desorption of NR molecules. Specifically, a portion of the strongly bound NR remained in the gel network after each cycle, occupying a fraction of the active binding sites and thereby reducing the available capacity for subsequent adsorption. Despite this gradual decrease, the CD–gel maintained over 60% of its initial adsorption capacity after three cycles, demonstrating acceptable reusability and structural stability for practical applications.

## 4. Conclusions

In summary, a polycarboxyl fluorescent composite hydrogel (CD–gel) with dual functions for Cr(VI) fluorescence detection and cationic dye NR adsorption was developed. Guided by the concept of “functional differentiation of groups”, CD-NH_2_ hydrothermally synthesized from CMCS were covalently incorporated into the gel network, endowing the material with fluorescence responsiveness while participating in Schiff base crosslinking. OSA acted as an aldehyde crosslinker and rapidly reacted with CMCS and CD-NH_2_ in aqueous solution at room temperature, forming a honeycomb-like mesoporous network with a BET surface area of 61.2 m^2^ g^−1^. FTIR and XPS results support the formation of the Schiff base crosslinked network and indicate that carboxyl groups were largely retained after crosslinking. The resulting gel was negatively charged over the pH range of 3–11, which favors electrostatic interactions with cationic species. Benefiting from these structural features, CD–gel exhibited sensitive fluorescence quenching toward Cr(VI), with a detection limit of 0.226 μM, a linear range of 0–80 μM, a response time of about 15 s, and good selectivity and anti-interference ability. Meanwhile, the retained carboxyl sites enabled rapid capture of NR through electrostatic attraction, hydrogen bonding, and n–π interactions, reaching adsorption equilibrium within about 30 min. The adsorption followed pseudo-second-order kinetics and the Langmuir isotherm equation (R^2^ = 0.9877), with a theoretical maximum adsorption capacity of 739.13 mg g^−1^, and was a spontaneous and endothermic process. Furthermore, the CD–gel maintained over 60% of its initial capacity after three consecutive adsorption–desorption cycles, demonstrating satisfactory reusability and structural stability. This study provides a group-engineering approach for designing multifunctional hydrogels, and the resulting CD–gel shows potential for pollutant detection and removal in water treatment.

## Figures and Tables

**Table 1:** The isotherm parameters for the adsorption of NR onto CD–gel.

Isotherm Model	Parameter	NR
Freundlich	*K*_F_ (mg g^−1^)	77.43
	*b*_F_	2.61
	*R* ^2^	0.9215
Langmuir	*Q*_m_ (mg g^−1^)	739.13
	*K*_L_ (L mg^−1^)	0.0220
	*R* ^2^	0.9877
	*K_T_* (L mg^−1^)	0.292
Temkin	*b_T_* (J mol^−1^)	16.46
	*R* ^2^	0.9800

**Table 2:** Kinetic parameters for the adsorption of NR on CD–gel.

Type	Parameter	NR
Pseudo-first-order	*k*_1_ (min^−1^)	0.1049
	qe-cal (mg g^−1^)	619.97
	*R* ^2^	0.9927
Pseudo-second-order	k_2_ (g mg^−1^ min^−1^)	0.000226
	qe-cal (mg g^−1^)	687.3
	qe-exp (mg g^−1^)	644.204
	*R* ^2^	0.9915
Intraparticle diffusion (Stage I)	*k_i d_*_1_ (mg g^−1^ min^−1/2^)	110.31
	*C*_1_ (mg/g)	26.08
	*R*_1_^2^	0.981
Intraparticle diffusion (Stage II)	*k_i d_*_2_ (mg g^−1^ min^−1/2^)	11.60
	*C*_2_ (mg/g)	517.5
	*R*_2_^2^	0.945

**Table 3:** Thermodynamic parameters for the adsorption of NR on CD–gel.

ΔH°	ΔS°	ΔG° (kJ mol^−1^)		
(kJ mol^−1^)	(J mol^−1^ k^−1^)	288 K	308 K	338 K
7.84	53.89	−7.68	−8.76	−10.37

## Data Availability

The original contributions presented in this study are included in the article/Supplementary Material. Further inquiries can be directed to the corresponding author.

## Supplementary Materials

The following supporting information can be downloaded at: https://www.mdpi.com/article/10.3390/ma19214750/s1, Table S1: Comparison of neutral red adsorption capacities of different adsorbents.
