# Supplementary material for: Polycarboxyl Fluorescent Hydrogel Prepared via Rapid Co-Crosslinking of Amino Carbon Dots and Composite Polysaccharide for Cr(VI) Detection and Efficient Adsorption of Neutral Red
Source: Materials (Basel). 2026 Nov 9;19(21):4750. doi: 10.3390/ma19214750 (PMC13765340; doi:10.3390/ma19214750)
Supplement: Supplementary file 1 [file materials-19-04750-s001.zip › materials-4627722-supplementary.pdf]

Polycarboxyl Fluorescent Hydrogel Prepared via Rapid Co-Crosslinking of Amino Carbon Dots and Composite Polysaccharide for Cr(VI) Detection and Efficient Adsorption of Neutral Red

Wang yichao[a] Qin chunrong[b] Luo rui[b] Zeng sihua[a]*

Guangxi Key Laboratory of Calcium Carbonate Resources Comprehensive Utilization, College of Materials and Chemical Engineering, Hezhou University, Hezhou, 542899, China.

*** Corresponding author.**

E-mail address: 202211002@hzxy.edu.cn (S. Zeng)

S1. Materials and Methods

S1.1 Adsorption Kinetic Analysis

To investigate the adsorption kinetic process, the adsorption data were analyzed using pseudo-first-order (PFO), pseudo-second-order (PSO), and intra-particle diffusion equations via non-linear regression. The kinetic equations are expressed as follows:

Pseudo-first-order equation:

$$Q_t = Q_e(1-e^{-k_1 t}) \quad \text{(S1)}$$

Pseudo-second-order equation:

$$Q_t = k_2 Q_e^2 t/(1-k_2 Q_e t) \quad \text{(S2)}$$

Intra-particle diffusion equation:

$$Q_t = k_{id}\, t^{0.5} + C \quad \text{(S3)}$$

Where Q_t (mg g^{-1}) represents the adsorption capacity at time t (min); k_1 (min^{-1}), k_2 (g mg^{-1} min^{-1}), and k_{id} (mg $g^{-1}\cdot min^{-1/2}$) denote the rate constants for the PFO, PSO, and intra-particle diffusion equations, respectively; and C is the constant associated with the boundary layer effect.

S1.2 Adsorption Isotherm Analysis

The Langmuir, Freundlich, and Temkin isotherm equations were applied to evaluate the effect of initial concentration on the adsorption process via non-linear regression. The isotherm equations are given below:

Langmuir equation:

$$Q_e = Q_m K_L C_e/(1+K_L C_e) \quad \text{(S4)}$$

Freundlich equation:

$$Q_e = K_F\, C_e^{b_F} \quad \text{(S5)}$$

Temkin equation:

$$Q_e = RT/b_T * \ln(a_T C_e) \qquad (S6)$$

Where Q_m (mg g^{-1}) is the maximum theoretical adsorption capacity; K_L(L mg^{-1}) is the Langmuir adsorption constant; K_F is the Freundlich adsorption constant; b_F (or 1/n) is a constant related to adsorption intensity; a_T (L mg^{-1}) and b_T(J mol^{-1}) are Temkin isotherm constants; and R (8.314 J mol^{-1} K^{-1}) is the ideal gas constant.

S1.3 Adsorption Thermodynamic Analysis

To better understand the influence of temperature on the adsorption process, the thermodynamic parameters were calculated based on the dimensionless thermodynamic equilibrium constant (Keq°). According to the methodology described by Tran et al. (2021), the equilibrium constants of the Henry, Freundlich, and Temkin models were not applied due to their unit limitations or lack of a true thermodynamic equilibrium constant. Therefore, Keq° was derived from the Langmuir equilibrium constant (KL, L·mg^{-1}) obtained at different temperatures, using the following conversion equation:

$$Keq° = K_L \times 10^3 \times C° / \gamma \qquad (S7)$$

Where C° is the standard concentration of the adsorbate (1 mol·L^{-1}) and γ is the activity coefficient (assumed to be unity in this study). The van't Hoff equation (Eq. 2) and the Gibbs free energy equation (Eq. 3) were then employed:

$$\ln Keq° = \Delta S° / R - (\Delta H° / R) \times (1/T) \qquad (S8)$$

$$\Delta G° = \Delta H° - T\Delta S° \qquad (S9)$$

Where ΔS° (J·mol^{-1}·K^{-1}) is the standard entropy change, R (8.314 J·mol^{-1}·K^{-1}) is the universal gas constant, ΔH° (kJ·mol^{-1}) is the standard enthalpy change, T (K) is the absolute temperature, and ΔG° (kJ·mol^{-1}) is the standard Gibbs free energy change. The values of ΔH° and ΔS° were determined from the slope and intercept of the linear plot of ln Keq° versus 1/T, respectively.

Table S1. Comparison of neutral red adsorption capacities of different adsorbents.

Adsorbent	Q_m (mg g^{-1})	Reference
Magnetic graphene oxide-containing chitosan-sodium alginate hydrogel beads	44.654	[37]
polyaniline–zirconium phosphoborate (PANI–ZrPB) nanocomposite	56.0	[38]
Cu(II) metal–organic gel	650.32	[39]
three-dimensional magnetic sulfur/nitrogen co-doped reduced graphene oxide nanomaterials	877.19	[40]
TAPT-HMIPA-COF	429	[41]
metal-organic framework	243.9	[42]
functionalized microcrystalline cellulose	83.2	[43]
nitrogen- and oxygen-containing GO-based composite	101.32	[44]
shock-absorbing filler	121.21	[45]
CDs-gel	739.13	This work

37. Ma, J.; Zhang, M.; Ji, M.; Zhang, L.; Qin, Z.; Zhang, Y.; Gao, L.; Jiao, T., Magnetic graphene oxide-containing chitosan-sodium alginate hydrogel beads for highly efficient and sustainable removal of cationic dyes. *International Journal of Biological Macromolecules* **2021,** 193, 2221-2231.

38. Kaushal, S.; Badru, R.; Kumar, S.; Kaur, H.; Singh, P., Efficient Removal of Cationic and Anionic Dyes from Their Binary Mixtures by Organic–Inorganic Hybrid Material. *Journal of Inorganic and Organometallic Polymers and Materials* **2018,** 28, (3), 968-977.

39. Wu, Q.; He, L.; Jiang, Z. W.; Li, Y.; Zhao, T. T.; Li, Y. H.; Huang, C. Z.; Li, Y. F., One-step synthesis of Cu(II) metal–organic gel as recyclable material for rapid, efficient and size selective cationic dyes adsorption. *Journal of Environmental Sciences* **2019,** 86, 203-212.

40. Rong, Y.; Huang, Y.; Jin, P.; Yang, C.; Zhong, Z.; Dong, C.; Liang, W., Highly efficient removal of cationic, anionic and neutral dyes by hierarchically porous structured three-dimensional magnetic sulfur/nitrogen co-doped reduced graphene oxide nanohybrid. *Journal of Water Process Engineering* **2020,** 37, 101345.

41. Rastegari, F.; Asghari, S.; Mohammadpoor-Baltork, I.; Sabzyan, H.; Tangestaninejad, S.; Moghadam, M.; Mirkhani, V., A pH-dependent and charge selective covalent organic framework for removal of dyes from aqueous solutions. *Journal of Hazardous Materials* **2024,** 476, 135075.

42. Ghomshehzadeh, S. G.; Nobakht, V.; Pourreza, N.; Mercandelli, P.; Carlucci, L., A new pillared Cd-organic framework as adsorbent of organic dyes and as precursor of CdO nanoparticles. *Polyhedron* **2020,** 176, 114265.
43. Bai, H.; Chen, J.; Zhou, X.; Hu, C., Single and binary adsorption of dyes from aqueous solutions using functionalized microcrystalline cellulose from cotton fiber. *Korean Journal of Chemical Engineering* **2020,** 37, (11), 1926-1932.
44. Liao, C.; Liu, Y.-P.; Lan, X.-W.; Jiang, X.-Y.; Liu, G.-C.; Yu, J.-G., Construction of a novel nitrogen- and oxygen-containing GO-based composite with specific adsorption selectivity. *Journal of Environmental Chemical Engineering* **2021,** 9, (1), 104952.
45. Samal, P. P.; Qaiyum, M. A.; Dutta, S.; Mohanta, J.; Dey, B.; Dey, S., Towards a circular economy: chemical packaging waste as a promising scavenger for Neutral red from water and wastewater. *International Journal of Environmental Science and Technology* **2023,** 20, (11), 12533-12544.
